# My patient might be depressed – can I still screen for MCI? Exploring cognitive performance on the MoCA in older people screened for depressive symptoms with the PHQ-9

**DOI:** 10.1186/s12877-025-06004-6

**Published:** 2025-05-24

**Authors:** Sophia Bösl, Petra Scheerbaum, Elmar Graessel, Christian Kessler, Julia-Sophia Scheuermann

**Affiliations:** 1 Centre for Health Services Research in Medicine, Department of Psychiatry and Psychotherapy, Uniklinikum Erlangen, Friedrich-Alexander-Universität Erlangen-Nürnberg (FAU), Schwabachanlage 6, D-91054 Erlangen, Germany; 2 Department of Internal and Nature-Based Therapies, Immanuel Hospital Berlin, D-14109 Berlin, Germany

**Keywords:** MoCA, Depressive symptoms, Cognitive performance, Older people

## Abstract

**Objective:**

The aim of this study was to compare the Montreal Cognitive Assessment (MoCA) performances of people who report no, subclinical, and clinical symptoms of depression.

**Methods:**

Data was collected for the randomized controlled trial BrainFit-Nutrition. A secondary data analysis of 1,111 participants (age ≥ 60 years; *M* = 68.4 years; 55.1% female) was performed. Depressive symptoms were assessed with the Patient Health Questionnaire-9 (PHQ-9), cognitive performance was assessed via the MoCA. Performance differences were tested with Kruskal-Wallis tests. Two sensitivity analyses were conducted, one with data from people with MCI and one with the original item structure of the MoCA.

**Results:**

No differences were found in the MoCA total score or in visuospatial, executive functioning, attention, memory, or orientation subscores between individuals with no, subclinical, or clinical symptoms of depression. A sensitivity analysis also showed no differences.

**Conclusion:**

Cognitive screening with the MoCA seems to be robust against depression and could therefore be used to screen for MCI regardless of depression level.

**Trial registration:**

The study was prospectively registered at the International Standard Randomized Controlled Trial Number Registry on 23/11/2021 (ISRCTN 10560738).

**Supplementary Information:**

The online version contains supplementary material available at 10.1186/s12877-025-06004-6.

## Introduction

The Montreal Cognitive Assessment (MoCA) [1] is used globally to screen for Mild Cognitive Impairment (MCI), as it has high sensitivity and specificity and can be administered easily and quickly [2]. Detection of MCI is essential for early treatment [3], as MCI is a prodromal stage to dementia [4, 5] and describes the decline in a person’s cognitive functioning as they transition from normal aging to dementia [6]. Thus, people with MCI are at significantly higher risk of developing dementia than cognitively healthy people [4]. However, it seems that not only MCI but also affective disorders, e.g. depression, have an impact on participants’ cognitive performance on the MoCA, at least in a clinical setting [7].

Depression is a mental disorder affecting over 300 million people of all ages worldwide [8]. In the older population (people 60 years of age and older), the prevalence is estimated to range from 17.5 to 31.7% [9]. Amongst other symptoms, impaired cognitive functioning, e.g. memory difficulties or limited concentration, can be present in people with depressive disorders [10]. Therefore, the distinction between depressive symptoms and early signs of dementia is complex, especially for older people [11], but it is clinically important. Depressive symptoms are assumed to be a risk factor for dementia [11–15] and can also emerge during the prodromal stage of dementia [11, 13].

Regarding specific dimensions of cognitive functioning, lower performance has been reported in memory [16], language [16–18], and executive as well as visuospatial functioning [16, 18–20] in older people with depression compared with non-depressed controls. People with MCI and depression seem to perform worse on items that measure visuospatial, memory, and executive functioning [21] compared with people with MCI without depression. Additionally, global cognitive functioning as well as memory, semantic fluency, and processing speed seem to decline more quickly in people with MCI and chronic subsyndromal, i.e. subclinical, depressive symptoms compared with people with MCI and no depressive symptoms [22].

In order to avoid false positive results in screened patients, it is necessary to know whether there are differences in the MoCA performance of older people with and without symptoms of depression so that the results can be interpreted accordingly. However, no information about potential differences in performance is available for the German version of the MoCA. It is therefore necessary to investigate whether differences in performance, as assessed with the total score and subscores from the German version of the MoCA, can be observed in people with and without depressive symptoms.

On the basis of previous research [16, 17, 20, 21], we hypothesized that MoCA subscores for memory, language, executive functioning, and visuospatial functioning as well as the total score would differ across older people with symptoms of clinical depression, symptoms of subclinical depression, and no depressive symptoms.

## Methods

### Design and sample

The secondary analysis was based on the screening data from the randomized controlled trial BrainFit-Nutrition. The study design was previously published as a study protocol [23].

**Fig. 1:**
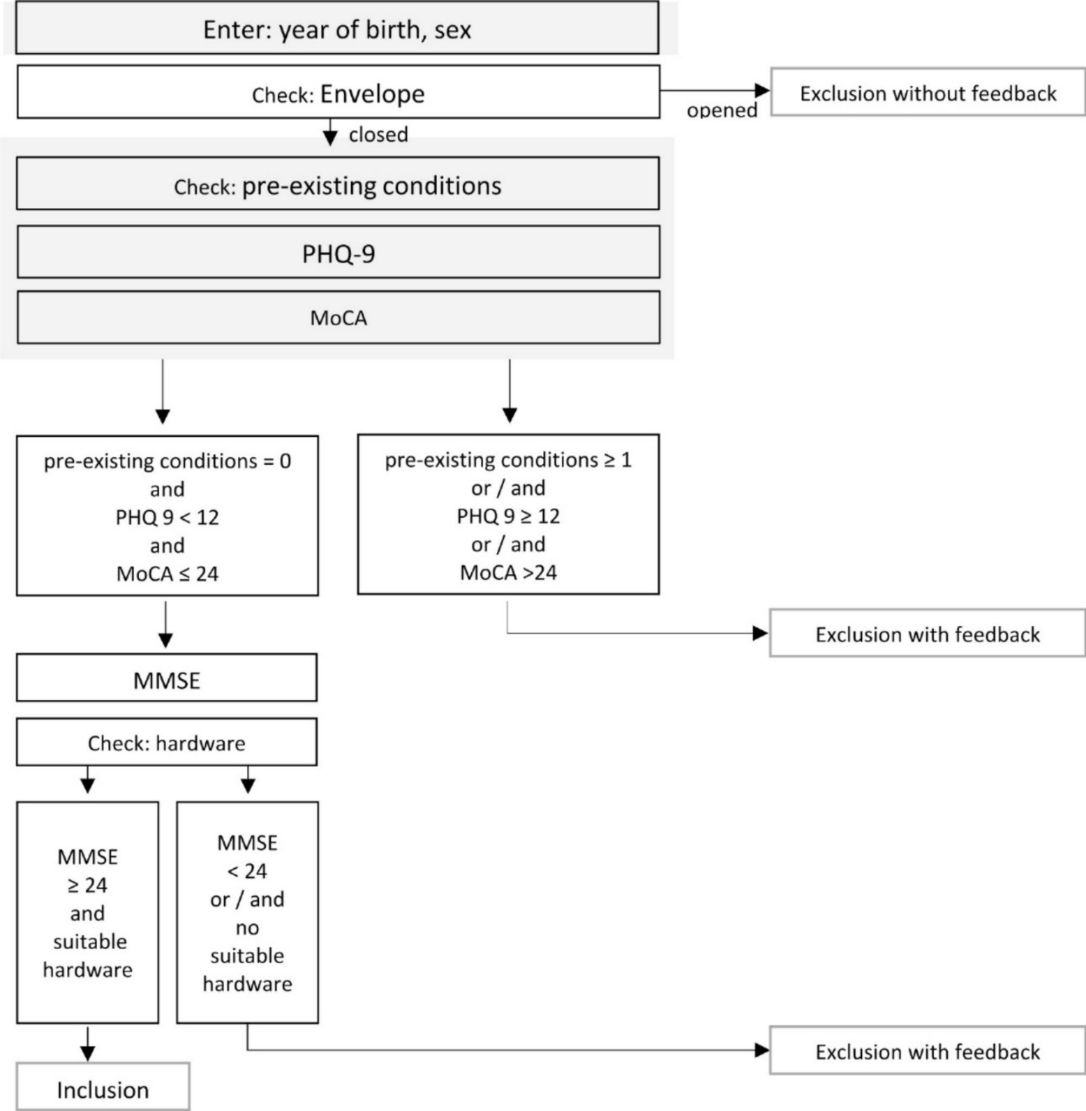
Screening procedure for BrainFit-Nutrition Note. PHQ-9, Patient Health Questionnaire-9, range 0–27; MMSE, Mini Mental Status Examination, range 0–30; MoCA, Montreal Cognitive Assessment, range 0–30. All information used for the present analysis is marked in grey

More than 1,100 people across Germany aged 60 and older were screened for MCI between January and September of 2022. For the following analysis, data from all participants who completed the first part of the screening were used (*N* = 1,111), thus avoiding a selection bias. In order to enable all residents of Germany to participate, the study was conducted completely remotely. The screening was performed as a video conference using the remote software samedi – certified from the German National Association of Statutory Health Insurance Physicians [24]. During the video conference, the interviewer entered the participants’ answers into the data collection system REDCap. If data entries were missing or invalid, the interviewer was immediately asked to correct the entry. In the screening for BrainFit-Nutrition study participation, the following information was assessed: completely blind or deaf; diagnosis of pre-existing somatic or psychiatric conditions that can cause cognitive impairment, e.g. psychosis, multiple sclerosis, Parkinson’s disease, or multiple strokes; symptoms of depression, assessed with the Patient Health Questionnaire-9 (PHQ-9) [25]; the presence of MCI, defined as a MoCA score > 24 [26–28]. Screening procedure for the study was terminated if participants met at least one of the exclusion criteria described in detail in the study protocol [23]. In Fig. 1, screening procedure is described and information of the participants used for the present analysis is shown. All procedures were approved by the Ethics Committee of the medical faculty of the Friedrich-Alexander-Universität Erlangen-Nürnberg (Ref.: 21–318_1-B). The study was prospectively registered at the International Standard Randomized Controlled Trial Number Registry on 23/11/2021 (ISRCTN 10560738).

### Instruments

#### PHQ-9

The PHQ-9 is a short questionnaire with nine items concerning different depressive symptoms within the last two weeks [25]. Each item can be answered on a scale ranging from 0 (“not at all“) to 3 (“nearly every day“), resulting in a maximum possible total score of 27. Originally, to determine the severity of the depressive episode, several cut-offs were established [25]: minimal (0–4), mild (5–9), moderate (10–14), moderately severe (15–19), and severe (20–27). However, a cut-off of ≥ 10 was found to have the highest sensitivity and specificity in distinguishing between people with and without symptoms of a major depressive episode [29]. As cognitive impairments may already occur in individuals with a subsyndromal depressive episode [22], in accordance with Kroenke et al. [25] we set the following cut-offs for depressive symptoms in the present study: “no– minimal” (0–4), “subclinical” (5–9), and “clinical” (≥ 10) symptoms of depression.

#### MoCA

The MoCA is a screening tool with high sensitivity and specificity [1], which can be used to differentiate between individuals with and without MCI. The instrument covers multiple cognitive domains, namely, visuospatial and executive functioning, speech, attention, memory, and orientation [1]. The items comprising each subscore can be found in Table 1. A total score of 30 can be achieved, and a score of ≤ 24 indicates MCI [25–28]. It is unclear whether verbal fluency is part of the executive or the language subscore [30, 31]. In Nasreddine et al. [1], verbal fluency was assigned to both subscores. However, the task for verbal fluency in the MoCA is more of an assessment of phonemic fluency and can therefore be assigned to the executive subscore, in contrast to semantic fluency [32]. For the current analysis, the decision was therefore made to assign the verbal fluency task to the executive subdomain.

The MoCA can be administered face-to-face or remotely. In the present study, the German adaption of the MoCA Version 8.1 was conducted remotely, based on the audio-visual conference protocol provided by the MoCA Clinic [33].

**Table 1:** Components of the MoCA subscores

Subscores^a^	Items	Range
Visuospatial	Clock-drawing test, cube-drawing test	0–4
Executive	Trail-making test, verbal fluency, verbal abstraction	0–4
Attention	Target detection task, serial subtraction task, digits forward and backward	0–6
Memory	Delayed recall task	0–5
Language	Confrontation naming task, repetition of sentences	0–5
Orientation	Questions about time and place	0–6

^*a*^Adapted from Nasreddine et al., 2005 [1]

### Statistical analysis

In order to examine group differences in MoCA subscores, a multivariate analysis of variance (MANOVA) was chosen and assumptions were tested. Outliers were found, but these values had to be kept in the data set, as no errors in recording could be assumed. Normal distributions could not be achieved for any of the dependent variables after the data were transformed by inversion (Shapiro-Wilks test α < 0.05). Further analyses were performed on the transformed data, and no multicollinearity between dependent variables was found (*r* <.80 in accordance with Field [34]). Additionally, no multivariate outliers were found (assessed by Mahalanobis distance, *p* >.001). Homogeneity of error variances could be reported for the executive, attention, memory, and orientation subscores (*p* >.05) but not for the visuospatial (*p* =.010) or speech (*p* <.001) subscores. There was homogeneity of covariances (Box’s test, *p* >.001).

As several assumptions for the MANOVA were not met, a considerably reduced validity of its results must be assumed. Thus, the non-parametric multivariate Kruskal-Wallis test was conducted on the untransformed data to test for group differences (α = 0.05).

Due to different approaches of item categorization and different sample characteristics in the abovementioned studies, sensitivity analyses were performed for better comparisons. Therefore, a multivariate Kruskal-Wallis test was conducted using only the data from people with MCI (*n* = 333) for better comparability with Lee et al. [21]. Additionally, for another sensitivity analysis, the Kruskal-Wallis test was performed with the original item structure of the MoCA, removing the previously applied categorizations (see Table 1), as seen in Dierckx et al. [20] and Blair et al. [17]. All statistical analyses were carried out using IBM SPSS Statistics 28.0.

## Results

### Main analysis

Table 2 presents descriptive statistics for age, sex, and cognitive functioning for the overall sample as well as grouped by the PHQ-9 categories. Most of the participants (94.7%) did not report pre-existing somatic or psychiatric conditions that could cause cognitive decline; 4.8% reported one pre-existing condition, and 0.5% reported two. The sample’s mean PHQ-9 score was 4.21 (*SD* = 3.84) with 63.2% of the participants reporting no symptoms of depression, 26.1% reporting subclinical symptoms, and 10.7% reporting clinical symptoms. The mean MoCA score was 25.03 (*SD* = 2.73). MCI, defined as MoCA ≤ 24 was found in 34.5% of the total sample (Table 2). The MoCA total score and each MoCA subscore for both the total sample and all three depression categories can be found in Table 3.

**Table 2:** Descriptive statistics for the sample

	Total Sample (*N* = 1,111)		No Symptoms PHQ-9 = 0–4 (*n* = 702)		Subclinical Symptoms PHQ-9 = 5–9 (*n* = 290)		Clinical Symptoms PHQ-9 ≥ 10 (*n* = 119)	
	m (*SD*)	n (%)	m (*SD*)	n (%)	m (*SD*)	n (%)	m (*SD*)	n (%)
Age	68.4 (6.4)		68.7 (6.5)		68.4 (6.9)		66.5 (6.0)	
Sex (female)		612 (55.1)		322 (45.9)		201 (69.3)		89 (74.8)
MCI (MoCA ≤ 24)		382 (34.4)		242 (34.5)		94 (32.4)		46 (38.7)

Abbreviations: m, mean; SD, standard deviation; %, frequency of the reference group (in brackets) for the respective category; PHQ-9, Patient Health Questionnaire-9, range 0–27; MoCA, Montreal Cognitive Assessment, range 0–30, scores ≤ 24 indicate Mild Cognitive Impairment

Kruskal-Wallis tests were performed to investigate possible differences in MoCA subscores between individuals with no, mild, or noticeable symptoms of a depressive episode. No significant differences were found in visuospatial functioning (*H*(2) = 0.053, *p* =.974), executive functioning (*H*(2) = 0.331, *p* =.848), attention (*H*(2) = 0.518, *p* =.772), speech (*H*(2) = 4.691, *p* =.096), memory (*H*(2) = 0.466, *p* =.792), orientation (*H*(2) = 0.076, *p* =.963), or the MoCA total score (*H*(2) = 1.196, *p* =.550) across the different depression categories.

**Table 3:** MoCA subscores

MoCA– subscores (max. score)	Total Sample (*N* = 1,111)	No Symptoms PHQ-9 = 0–4 (*n* = 702)	Subclinical Symptoms PHQ-9 = 5–9 (*n* = 290)	Clinical Symptoms PHQ-9 ≥ 10 (*n* = 119)
	m (*SD*)	m (*SD*)	m (*SD*)	m (*SD*)
Visuospatial (4)	3.29 (0.80)	3.27 (0.03)	3.33 (0.40)	3.29 (0.29)
Executive (4)	2.57 (0.90)	2.58 (0.03)	2.57 (0.05)	2.50 (0.09)
Attention (6)	5.34 (0.84)	5.36 (0.03)	5.32 (0.05)	5.30 (0.08)
Memory (5)	3.29 (1.47)	3.28 (0.06)	3.33 (0.09)	3.24 (0.14)
Language (5)	4.75 (0.54)	4.76 (0.02)	4.75 (0.03)	4.64 (0.06)
Orientation (6)	5.80 (0.49)	5.79 (0.02)	5.81 (0.03)	5.81 (0.04)
Total Score (30)	25.03 (2.73)	25.04 (0.11)	25.12 (0.15)	24.78 (0.27)

Abbreviations: m, mean; SD, standard deviation; PHQ-9, Patient Health Questionnaire-9, range 0–27; MoCA, Montreal Cognitive Assessment, range 0–30, scores ≤ 24 indicate Mild Cognitive Impairment

### Sensitivity analysis

The mean age of people with psychometrically defined MCI (MoCA total score ≤ 24; *n* = 333) was 70.9 years (SD = 7.09), and 51.7% were female. The MoCA total score and each MoCA subscore for people with no symptoms (68.2%), subclinical symptoms (26.3%), and clinical symptoms (5.7%) of depression as well as the results of the Kruskal-Wallis test can be found in Supplement 1. No significant differences were found in MoCA scores across the depression categories. Likewise, no significant differences were found when applying the original MoCA item structure. Item scores and results of the Kruskal-Wallis test can be found in Supplement 2.

## Discussion

The aim of this paper was to compare performance on the MoCA total score and subscores for people with no symptoms, subclinical symptoms, and clinical symptoms of depression.

This sample is comparable to other studies or prevalence rates in the population with respect to the frequency of depressive symptoms: In the present sample, the rate of depressive symptoms was higher in women than in men, in line with the review results of Eid et al. [35]. The gender ratio of 3:1 that was found in the German sample in the present study corresponds to the gender ratio in other high-income countries reported by Bormet et al. [36] as well as to the ratio found in a more diverse sample of older people reported in Padayachey et al. [37].

In contrast to previous studies using the MoCA [17, 20] or other cognitive tests [16, 21], no differences in performance could be found between people with and without depressive symptoms. Both Dierckx et al. [20] and Blair et al. [17] reported lower scores on participants’ overall test performance, especially on the visuospatial and executive tasks, for people with symptoms of depression compared with people without symptoms of depression. In the current study, no group differences were found in either the visuospatial or executive subscores. As neither previous study used subscores but instead compared performance on each MoCA item separately via t-tests, it could be assumed that differences in task performance may have remained undetected in the current study because subscores were analyzed. However, removing the categorization of the MoCA subscores in the second sensitivity analysis showed that focusing on the individual items did not change the results. Thus, the considerably smaller sample sizes in Dierckx et al. [20] and Blair et al. [17] may offer an alternative explanation for the differences that were found in their work but could not be found in the current study.

However, it should be emphasized that negative screening results for MCI in the MoCA do not necessarily indicate normal cognitive performance. Additionally, finding no differences in cognitive performance between people with no symptoms, subclinical symptoms, and clinical symptoms of depression does not imply that cognitive impairment in people with depression does not exist. Lee et al. [21] reported differences in cognitive performance between people with MCI and depression compared with people with MCI without depression in a sample with more than 3,000 participants. As they did not include participants who showed age- and education-appropriate cognitive performance, the findings from their study were limited to people who were already cognitively impaired. However, as seen in the sensitivity analysis in the present study, still no differences in test performance were found when using data only from people with MCI. It should be noted that most of the tests used in the study by Lee et al. [21] were part of neuropsychological test batteries that were designed to thoroughly test for cognitive impairment. These tests may therefore be more complex than the MoCA screening test and could be more sensitive for capturing cognitive deficits in people with depression. Wei et al. [16] also found that participants aged 60 and older with higher scores on the PHQ-9 had poorer performances in memory, language, and executive functioning. For the memory task, the Delayed Word Recall Test from the Consortium to Establish a Registry for Alzheimer’s Disease was used. For this task, the participants were asked to memorize 10 words (instead of five on the MoCA) and repeat them three times (instead of once on the MoCA). Additionally, executive functioning was measured with the Digit Symbol exercise from the Wechsler Adult Intelligence Scale– revised version, which considers psychomotor speed and learning [38].

In a meta-analysis, Semkovska et al. [39] found that, on tasks without time limits, no differences between people with depression and healthy controls could be found. As most of the tasks on the MoCA do not have time limits, factors such as psychomotor speed or processing speed are not part of the assessment [40]. To fully assess cognitive impairment in people with depression, it seems that more demanding neuropsychological instruments need to be used [41, 42].

This study showed that in the present sample, no differences in cognitive performance were found for people with no, subclinical, and clinical depressive symptoms as measured with the German MoCA. As the MoCA is used to screen for people with MCI, people with depressive symptoms might not necessarily be challenged by the MoCA items. Therefore, according to the findings of this secondary analysis, the MoCA can be used to screen for MCI for patients with depression.

### Limitations and strengths

This analysis has some limitations: First, the analysis was conducted as a secondary data analysis. Thus, it was limited by the context in which the sample was collected: Interested individuals were screened for MCI to recruit participants for the BrainFit-Nutrition intervention study. No information was collected on depressive episodes or clinical diagnoses of depressive disorders. Furthermore, depressive symptoms were measured via self-assessment, which could be influenced by response biases, such as social desirability. Second, cognitive performance was assessed only with screening instruments, and MCI was not clinically diagnosed. Third, data on education was not obtained during the screening process. Therefore, MoCA scores could not be corrected for level of education. Fourth, the digital setting has a limiting effect, as only people who were willing to be examined remotely via video conference took part in the screening process.

Nevertheless, there are also some strengths. First, the data are remarkably complete, as the participants were not able to skip questions because they were individually interviewed during the video consultation. Second, the data were collected from all over Germany to balance regional differences. Third, we used a validated screening instrument, the MoCA, to screen for MCI. The MoCA is widely used, thereby increasing comparability to other studies. Fourth, people with subclinical symptoms of depression were also included in the analysis. Fifth, an in-depth analysis of assumptions was conducted to obtain reliable results.

### Conclusions and future directions

In conclusion, people with depression can be screened for MCI with the MoCA without its score being significantly influenced by depressive symptoms. As these findings are based on a secondary analysis, prospective studies should be planned that can provide further evidence. For these future studies, more data from people with clinical signs of depression should be collected. Additionally, the use of several instruments to assess cognitive performance might be helpful to explain possible intra- and inter-individual differences or similarities in test performance.

## Electronic supplementary material

Below is the link to the electronic supplementary material.

Supplementary Material 1

## Data Availability

The data presented in this study are available upon reasonable request from the corresponding author. The data are not publicly available due to privacy.

## Abbreviations

MANOVA: Multivariate Analysis of Variance
MCI: Mild Cognitive Impairment
MMSE: Mini Mental Status Examination
MoCA: Montreal Cognitive Assessment
PHQ-9: Patient Health Questionnaire-9
