# Supplementary material for: My patient might be depressed – can I still screen for MCI? Exploring cognitive performance on the MoCA in older people screened for depressive symptoms with the PHQ-9
Source: BMC Geriatr. 2025 May 24;25:374. doi: 10.1186/s12877-025-06004-6 (PMC12102864; doi:10.1186/s12877-025-06004-6)
Supplement: Supplementary file 1 — Supplementary Material 1 [file 12877_2025_6004_MOESM1_ESM.docx]

**Supplement 1.** Sensitivity Analysis I – MCI Subsample

|  | No Symptoms  PHQ-9 = 0-4  (*n*= 227) | Subclinical Symptoms PHQ-9 = 5-9  (*n*= 87) | Clinical Symptoms PHQ-9 ≥ 10  (*n*= 19) |  |  |
| --- | --- | --- | --- | --- | --- |
| MoCA – subscores  (max. score) | m (*SD*) | m (*SD*) | m (*SD*) | χ^2^ (df) | *p* |
| Visouspatial (4) | 2.85 (0.06) | 3.00 (0.08) | 2.84 (0.24) | 2.04 (2) | .361 |
| Executive (4) | 2.17 (0.06) | 2.10 (0.09) | 1.84 (0.18) | 3.07 (2) | .215 |
| Attention (6) | 5.04 (0.06) | 4.92 (0.10) | 5.11 (0.13) | 0.64 (2) | .727 |
| Language (5) | 4.57 (0.04) | 4.54 (0.07) | 4.58 (0.14) | 0.30 (2) | .859 |
| Memory (5) | 2.04 (0.10) | 1.94 (0.14) | 2.32 (0.41) | 1.00 (2) | .607 |
| Orientation (6) | 5.66 (0.04) | 5.72 (0.06) | 5.79 (0.10) | 1.08 (2) | .582 |
| Total Score (30) | 22.33 (0.11) | 22.25 (0.17) | 22.47 (0.38) | 0.72 (2) | .698 |

*Abbreviations*: m, mean; SD, standard deviation; PHQ-9, Patient Health Questionnaire 9, range 0-27; MoCA, Montreal Cognitive Assessment, range 0-30.

**Supplement 2.** Sensitivity Analysis II – Categorization According to MoCA Worksheet

|  | No Symptoms PHQ-9 = 0-4  (*n* = 702) | Non-clinical Symptoms  PHQ-9 = 5-9  (*n* = 290) | Clinical Symptoms  PHQ-9 ≥ 10  (*n* = 119) |  |  |
| --- | --- | --- | --- | --- | --- |
| MoCA –  subscores  (max. score) | m (*SD*) | m (*SD*) | m (*SD*) | χ^2^ (df) | *p* |
| Visuospatial / Executive (5) | 3.69 (0.37) | 3.73 (0.05) | 3.71 (0.09) | 0.001 (2) | .999 |
| Naming (3) | 2.97 (0.01) | 2.96 (0.01) | 2.94 (0.03) | 1.378 (2) | .502 |
| Attention (6) | 5.36 (0.31) | 5.32 (0.05) | 5.30 (0.08) | 0.518 (2) | .772 |
| Language (3) | 2.28 (0.03) | 2.29 (0.04) | 2.21 (0.07) | 0.760 (2) | .684 |
| Abstraction (2) | 1.67 (0.02) | 1.67 (0.03) | 1.57 (0.05) | 4.232 (2) | .120 |
| Delayed recall (5) | 3.28 (0.06) | 3.33 (0.09) | 3.24 (0.14) | 0.466 (2) | .792 |
| Orientation (6) | 5.79 (0.02) | 5.81 (0.03) | 5.81 (0.04) | 0.076 (2) | .963 |

*Abbreviations*: m, mean; SD, standard deviation; PHQ-9, Patient Health Questionnaire 9, range 0-27; MoCA, Montreal Cognitive Assessment, range 0-30.
